# Non-Invasive Measurement of Adrenocortical Activity in Blue-Fronted Parrots (*Amazona aestiva*, Linnaeus, 1758)

**DOI:** 10.1371/journal.pone.0145909

**Published:** 2015-12-30

**Authors:** João C. P. Ferreira, Caroline J. Fujihara, Erika Fruhvald, Eduardo Trevisol, Flavia C. Destro, Carlos R. Teixeira, José C. F. Pantoja, Elizabeth M. S. Schmidt, Rupert Palme

**Affiliations:** 1 Department of Animal Reproduction and Veterinary Radiology, School of Veterinary Medicine and Animal Science, Unesp – Univ Estadual Paulista, Rubião Junior s/n, 18.618-970, Botucatu, Brazil; 2 Department of Veterinary Surgery, School of Veterinary Medicine and Animal Science, Unesp – Univ Estadual Paulista, Rubião Junior s/n, 18.618-970, Botucatu, Brazil; 3 Department of Veterinary Hygiene and Public Health, School of Veterinary Medicine and Animal Science, UNESP – Univ Estadual Paulista, Rubião Junior s/n, 18.618-970, Botucatu, SP, Brazil; 4 Department of Veterinary Clinics, School of Veterinary Medicine and Animal Science, Unesp – Univ Estadual Paulista, Rubião Junior s/n, 18.618-970, Botucatu, Brazil; 5 Department of Biomedical Sciences, Unit of Physiology, Pathophysiology and Experimental Endocrinology, University of Veterinary Medicine, Veterinärplatz 1, 2210, Vienna, Austria; John Hopkins University School of Medicine, UNITED STATES

## Abstract

Parrots kept in zoos and private households often develop psychological and behavioural disorders. Despite knowing that such disorders have a multifactorial aetiology and that chronic stress is involved, little is known about their development mainly due to a poor understanding of the parrots’ physiology and the lack of validated methods to measure stress in these species. In birds, blood corticosterone concentrations provide information about adrenocortical activity. However, blood sampling techniques are difficult, highly invasive and inappropriate to investigate stressful situations and welfare conditions. Thus, a non-invasive method to measure steroid hormones is critically needed. Aiming to perform a physiological validation of a cortisone enzyme immunoassay (EIA) to measure glucocorticoid metabolites (GCM) in droppings of 24 Blue-fronted parrots (*Amazona aestiva*), two experiments were designed. During the experiments all droppings were collected at 3-h intervals. Initially, birds were sampled for 24 h (experiment 1) and one week later assigned to four different treatments (experiment 2): Control (undisturbed), Saline (0.2 mL of 0.9% NaCl IM), Dexamethasone (1 mg/kg IM) and Adrenocorticotropic hormone (ACTH; 25 IU IM). Treatments (always one week apart) were applied to all animals in a cross-over study design. A daily rhythm pattern in GCM excretion was detected but there were no sex differences (first experiment). Saline and dexamethasone treatments had no effect on GCM (not different from control concentrations). Following ACTH injection, GCM concentration increased about 13.1-fold (median) at the peak (after 3–9 h), and then dropped to pre-treatment concentrations. By a successful physiological validation, we demonstrated the suitability of the cortisone EIA to non-invasively monitor increased adrenocortical activity, and thus, stress in the Blue-fronted parrot. This method opens up new perspectives for investigating the connection between behavioural disorders and stress in this bird species, and could also help in their captive management.

## Introduction

The Blue-fronted parrot (*Amazona aestiva*, Linnaeus 1758) is a monogamous species that lives in large groups. Because of their sociability, beauty, and ability to imitate human speech these birds are kept as pets in private households. They are also frequently present in large numbers in wild animal triage centres, and zoos [1–5]. Once kept in captivity these birds often develop a series of psychological and behavioural disorders, such as aggressiveness toward humans, feather plucking and screaming, and also self-mutilation, phobias, and stereotypies [6–10].

However, observations that Psittacine birds kept in the same environment display a large variation in the occurrence and severity of such disorders signalize that these problems could have a multifactorial aetiology. Besides environmental challenges the personality of a bird also matter [9,11]. For example, under chronic stress conditions, proactive birds seemed to be more prone to develop behavioural disorders than reactive ones [12]. Despite knowing that behavioural disorders in captive parrots have a multifactorial aetiology and that chronic stress is involved, little is known about their development mainly due to a poor understanding of the parrots’ physiology and the lack of validated methods to measure stress in these birds. [6–9]

Stress is an adaptive response that allows animals to cope with environmental challenges [13,14]. In vertebrates, the front-line hormones to overcome stressful situations are the glucocorticoids and catecholamines [15,16]. The main glucocorticoid in birds is corticosterone and its quantification provides information about adrenocortical activity [17,18], and has been used as an index of stress in birds [19,20]. However, in wild and/or small birds blood sampling is difficult, highly invasive and inappropriate for long-term monitoring of HPA activity [21,22]. Thus, a non-invasive method to measure steroid hormones is critically needed.

Several authors have reported a correlation between concentrations of plasma glucocorticoids and their metabolites in the faeces of mammals [23–25] or the droppings of birds [26]. However, species- and sex-specific differences in the types of formed glucocorticoid metabolites (GCM) result in a characteristic pattern of GCM present in the faeces of a given species [27,28]. Therefore, it is important to select an assay system capable of detecting most, or at least a relevant portion, of the respective GCM present in the faeces of the species investigated [27,28].

So far only test kits designed to measure blood corticosterone have been used to monitor concentrations of GCM in the droppings of parrots species, such as the Red-tailed parrot [29], the budgerigars (*Melopsittacus undulatus*) [30], and the Blue-fronted parrots [31]. In Red-tailed parrots an annual pattern of GCM concentrations was reported, but a physiological validation could not be performed [29] and in budgerigars only males were included in the validation tests [30].

Because in birds almost no native glucocorticoids are present in excreta, the applied kits worked due to antibody cross-reactions with GCM [27]. In several species, including the Blue-fronted parrots [31], blood kits have proved to be inadequate, as they did not show significant cross-reactions with the excreted faecal metabolites [32]. For this reason it is highly advisable to work with group-specific antibodies, which recognise a group of metabolites rather than a specific steroid and therefore have the benefit of detecting smaller increases of GCM [27]. Whatever assay is used to measure GCM, a sound validation of the method is mandatory [33–35].

The aim of this study was to perform a physiological validation of an enzyme immunoassay to measure glucocorticoid metabolites in droppings of captive Blue-fronted parrots (*Amazona aestiva*).

## Material and Methods

### Ethics statement

This study was conducted from August to October 2010 at the Centre for Medical Research and Wildlife (CEMPAS) of the School of Veterinary Medicine and Animal Science (FMVZ)—Universidade Estadual Paulista (UNESP) in Botucatu, Sao Paulo, Brazil (22°53′S; 48°26′W), in strict accordance with the recommendation of the Conselho Nacional de Controle de Experimentação Animal (CONCEA—Brazil). The study was also approved by the Committee on the Ethics of Animal Experiments of the FMVZ—UNESP (Permit number: 192/2008—CEUA).

### Animals

The study population comprised 24 adult (12 of each sex), clinically healthy Blue-fronted parrots (*Amazona aestiva*), rescued from illegal traffic, weighing 411 ± 51 g (mean ± standard deviation). All birds had been housed at CEMPAS for at least six months in two wide (53 m^2^) and adjacent aviaries with indoor and outdoor compartments before the beginning of the pre-experimental phase. During the pre- and experimental phases the birds were housed in individual cages of 100 x 50 x 50 cm (height, width and depth, respectively). The cages were placed in an outdoor facility, provided with a covered area, wind protection and exposed to natural light variations. Birds were fed specific pelleted food (Papagaio Mix^®^, Biotron, Rio Claro, Brazil) and had access to tap water *ad libitum*. Birds were kept in the same facility during all experiments and were able to witness all the procedures the others were subjected to.

During the experimental periods (experiments 1 and 2) all droppings excreted by each animal were collected at 3-h intervals (from 5 a.m. of the first day until 8 a.m. of the following day). In order to facilitate dropping collection, the bottom of the cages was lined with plastic sheet. All samples were immediately deposited in cryotubes, identified, and stored at -20°C.

### Experimental procedures

#### Pre-experimental phase

During thirty days before starting the experiments, the birds were adapted to the main conditions related to the experimental settings. Once a week all birds were subjected to manual restraint and injection of 0.2 mL of saline (0.9% NaCl—Sanobiol^®^, São Paulo, Brazil) in the pectoral muscle at 8 a.m. Afterwards, the birds were monitored for 48 h and all voided droppings were collected at 3-h interval.

#### Experiment 1. Diurnal variation of glucocorticoid metabolites (GCM)

After the 30-day adaptation period, the bird droppings were sampled every 3 h for 27 h to establish undisturbed baseline GCM concentrations.

#### Experiment 2. Physiological validation of an enzyme immunoassay to measure GCM

One week after experiment 1 the birds were randomly assigned to four groups of six animals each (three males and three females) and underwent four treatments in a cross-over design as follows: *Control*: the birds were kept undisturbed, *Saline*: 0.2 mL of saline (0.9% NaCl—Sanobiol, São Paulo, Brazil), *Dexamethasone*: 1 mg/kg of dexamethasone (Azium^®^, Coopers, Cotia, Brazil) [36], and *ACTH*: 25 IU diluted in 0.2 ml of saline (porcine ACTH, A6303, Sigma Aldrich, Brazil) [37,38].

All treatments were given as 0.2 mL injections in the pectoral muscle between 7:55 and 8:05 a.m. Before starting the treatments, all solutions (saline, dexamethasone and ACTH) were previously prepared in the syringes. Two persons were especially trained and familiarized with the procedures. One person performed the injections and the other restrained the birds. As the birds were also familiar with the procedures (due to the 30-day adaptation period described above), they did not experience a novel situation and displayed no signs of stress at seeing the others being captured. Moreover, during the whole study, including the adaptation period, the same two persons were responsible for providing the pelleted food and cleaning the cages, consequently the birds were accustomed to their presence. These procedures were repeated four times so that all animals were subjected to all treatments. There was a week interval between each treatment.

### Steroid extraction and quantification

Steroids were extracted using the methanol-based procedure described by Palme [39]. Initially the droppings were lyophilized and kept stored at -20°C. Each interval sample was thoroughly homogenized and an aliquot of 0.05 g was weighed and shaken for 30 min on a multivortex with one mL of 80% methanol. The suspension was then centrifuged at 500 g for 20 min and the supernatant was recovered. An aliquot (0.5 mL) of the supernatant was transferred into new vials and evaporated at 50°C for 14 h. After evaporation the dried extracts were stored at room temperature in dark boxes for 15–30 days and then kept at -20°C until assayed. One day before the GCM analyses the dried extracts were re-diluted in 0.5 mL of 80% methanol.

Glucocorticoid metabolites were quantified in an aliquot of the extract (diluted 1:10 in assay buffer) using a group-specific cortisone enzyme immunoassay (EIA) first described and validated in chicken by Rettenbacher et al. [32]. The EIA was performed on microtitre plates using the double antibody technique and biotinylated steroids were used as label. The antibody was raised in a rabbit immunised against cortisone-21-HS:BSA. The cross-reactivity of the antibody was as follows: cortisone (4-pregnene-17α,21-diol-3,11,20-trione), 100%; 4-androstene-3,11,17-trione, 30%; 5α-androstane-3,11,17-trione, 20%; 4-pregnene-11ß,17,20,21-tetrol-3-one, 9%; 4-pregnene-3,20-dione, 2.3%; 5ß-androstane-3,11,17-trione, 2.2%; 4-pregnene-11ß,21-diol-3,20-dione, 1.8%; 4-androstane-3,17-dione, 0.9%; 5α-androstane-3,17-dione, 0.5%; 5α-androstane-3α-ol-11,17-dione; 5α-androstane-3ß-ol-11,17-dione; 5ß-androstane-3α-ol-11,17-dione had cross-reactions below 0.1%.

Standards and samples were incubated in duplicate with label (100 μL) and antibody (100 μL) overnight at 4°C. After incubation, the plates were washed with 0.02% Tween 20 solution (Merck, Darmstadt, Germany 822184) and blotted dry before 250 μl of streptavidin horseradish peroxidase conjugate (4.2 mU, Art. No. 1089153, Boehringer, Mannheim, Germany) were added to each well. Plates were left in the dark on stirring tables for 45 min at 4°C. After the next washing step, 250 μl of tetramethylbenzidine (69.4 nmol/well; Art. No. 1089153, Boehringer, Germany) were added to each well and the plates were again incubated at 4°C. After 45 min the enzymatic reaction was stopped with 50 μl of 1 mol/L sulphuric acid. Absorbance was measured at a wavelength of 450 nm (reference filter: 620 nm) on an automatic plate reader (BioTek EL808; Szabo-Scandic, Vienna, Austria).

Intra- and inter-assay coefficients of variation were 5.3% and 7.7%, respectively, and the sensitivity of the assay was 1.4 ng/g droppings. All samples were assayed in duplicate. Concentrations of GCM were expressed as nanograms per gram of droppings dry matter.

### Statistics

GCM concentrations were log transformed before analysis to achieve normality. A repeated measures model (PROC MIXED, SAS Institute, 2011) was used to compare GCM concentrations between treatments (control, saline, dexamethasone or ACTH), sexes, and time points. Interaction terms between explanatory variables were included in the model and remained if significant. An auto-regressive covariance structure provided the best fit to model the correlation between the repeated measurements within the same animals. The Tukey´s test was used to adjust the *P*-values resulting from multiple comparisons. Statistical significance was set for *P* < 0.05.

## Results

### Experiment 1

**Fig 1:**
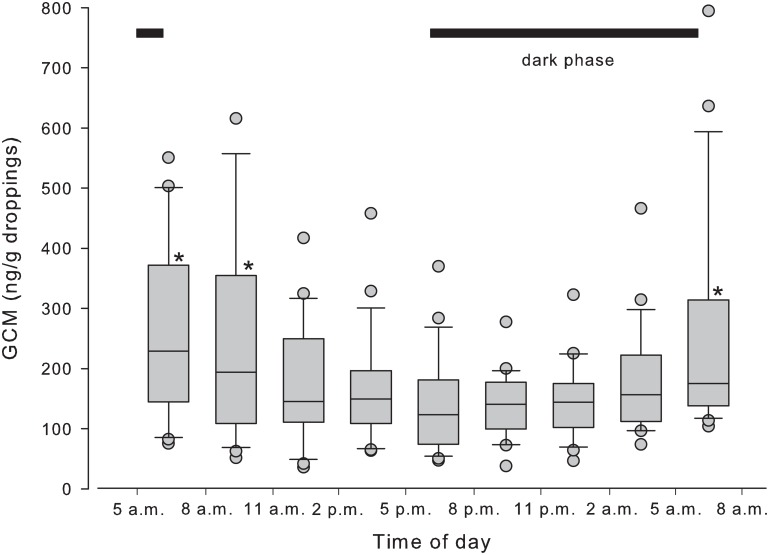
Boxplots of diurnal variation of concentrations of glucocorticoid metabolites (GCM; ng/g droppings) of Blue-fronted parrots (*Amazona aestiva*), kept under natural light variation (sunrise: 06:23 a.m.—sunset: 06:03 p.m). Respective median values are showed as a line in the boxes. Lower and upper boundaries of the boxes indicate the 25^th^ and 75^th^ percentiles, respectively. Whiskers above and below the box indicate the 90^th^ and 10^th^ percentiles. Outliers are show as points. Asterisks beside the box indicate significant differences compared with the 5 to 8 p.m. interval (*P* ≤ 0.005).

In experiment 1, droppings of all birds were available in all collection intervals. Baseline GCM concentrations showed a wide variation (31 to 1,905 ng/g droppings). No effect of gender (*P* = 0.38), but a statistically significant effect of time on GCM concentration was found (*P* < 0.001). The highest and lowest concentrations were observed in droppings voided from 5 to 11 a.m., and from 5 to 8 p.m., respectively (Fig 1).

### Experiment 2

Of all samples that could be collected (864), 844 (= 97.7%) were available. The analysis of GCM concentrations showed a significant effect of sex (*P* = 0.03). When the data of males and females were submitted to rmGLM significant effects of time (*P* < 0.001), treatment (*P* < 0.001), and also an interaction between time and treatment were observed (*P* < 0.001). Despite the detected general sex effect, there were no differences in GCM concentrations of males and females when compared for each observational time point (*P* = 0.9). The sex effect was restricted to two treatments as described below.

**Fig 2:**
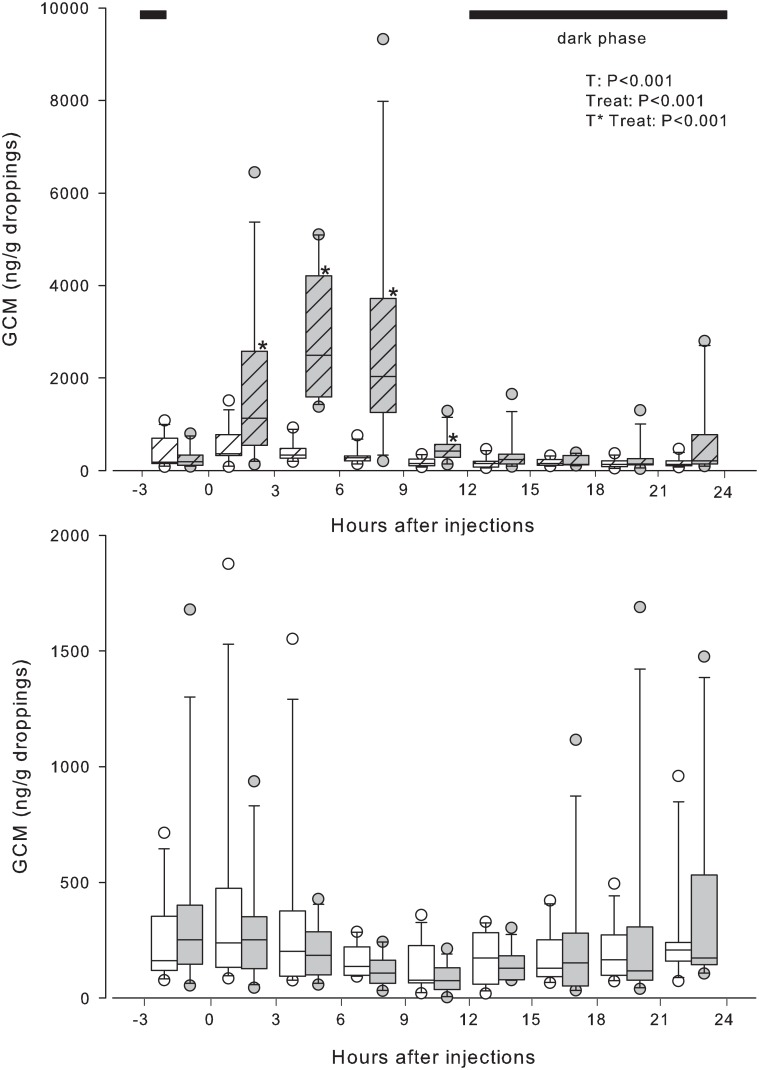
Boxplots (description see Fig 1) of concentrations of glucocorticoid metabolites (GCM; ng/g droppings) of female Blue-fronted parrots (*Amazona aestiva*), kept under natural light variation (sunrise: 06:02 ± 0:11 a.m.—sunset: 06:09 ± 0:03 p.m.–mean ± standard deviation), after an injection of saline solution (0.9% NaCl; white boxes), dexamethasone (1 mg/Kg; white striped boxes) and adrenocorticotropic hormone (25 IU of ACTH; gray striped boxes). The control group was kept in the same room but remained undisturbed (gray boxes). Please note the different y-axis scale in the upper (Dexamethasone; ACTH) and lower (Saline; Control) panels. Asterisks beside the gray striped boxes indicate significant differences compared with the same interval of the control group (*P* < 0.01). The P values of time (T), treatments (Treat) and the interaction time*treatments (T*Treat) are shown.

**Fig 3:**
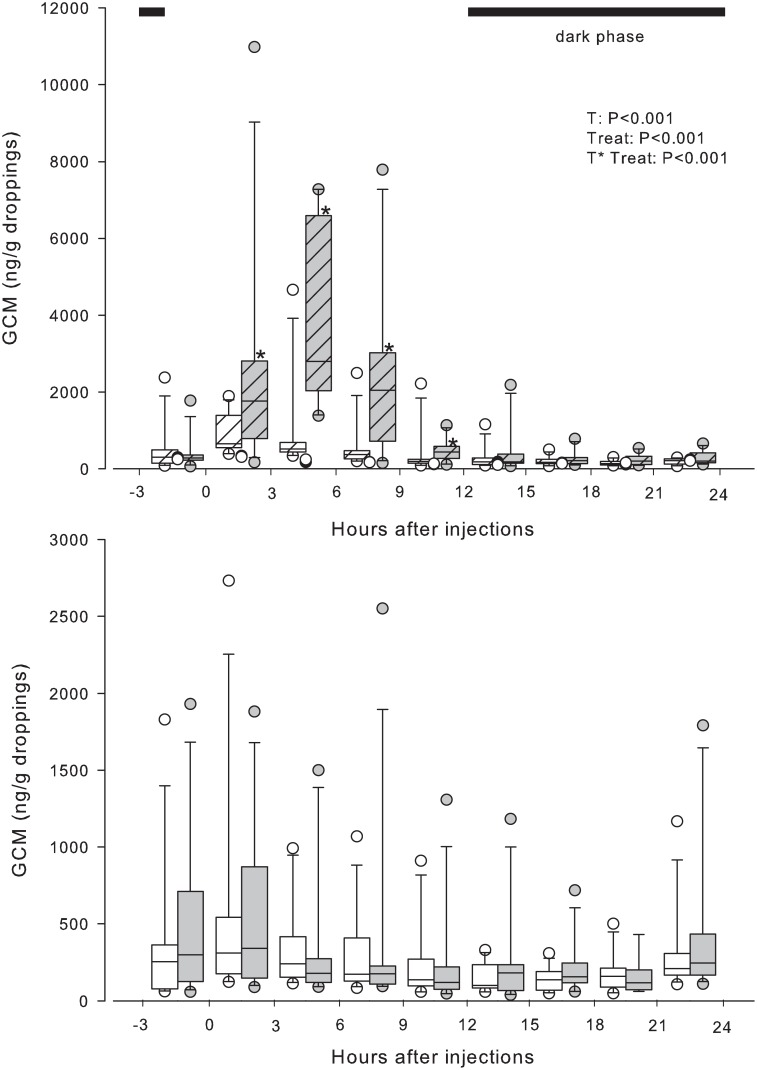
Boxplots of concentrations of glucocorticoid metabolites (GCM; ng/g droppings) of male Blue-fronted parrots (*Amazona aestiva*). For further details please see legend of Fig 2.

As observed in experiment 1, GCM concentrations also showed a marked time effect (*P* < 0.001) in the undisturbed control treatment in females. Droppings voided between 5 to 8 p.m. (9 to 12 h interval–Fig 2) had the lowest GCM concentration of the day. Afterwards concentrations started to increase and returned to initial values. In males, no time effect was detected in the control treatment (*P > 0*.*05*; Fig 3). Following the saline injection, GCM concentrations in females and males were similar to those observed in the control treatment (*P* > 0.5). Dexamethasone administration led to a transient increase in GCM in males, between 0 to 3 h after treatment (*P* = 0.004), followed by a return to pre-treatment concentrations after 3 h (Fig 3). This effect was not observed in female birds. When comparing the baseline concentrations derived from control and saline treatments, no significant effect was observed after dexamethasone administration.

For both sexes, the administration of ACTH led to an immediate increase in GCM in the samples collected from 0 to 3 h after treatment (*P <* 0.001; Figs 2 and 3). GCM concentrations increased about 13.1-fold (median) at the peak (after 3–9 h) and dropped to pre-treatment concentrations after 9 h. Considerable differences were found between individuals concerning initial and maximal concentrations (range 67 to 1,775 ng/g, and 1,538 to 28,124 ng/g, respectively). However, there was a good synchrony between the timing of peaks of different individuals (8%—3h; 50%—6h, 42%—9h) after treatment. Compared to the baseline values derived from the control treatment, GCM concentrations were significantly higher at 0 to 3, 3 to 6, 6 to 9 (*P* < 0.001), and 9 to 12 h intervals (*P* < 0.02) after the ACTH injection.

## Discussion

In our cross-over study with a high number of animals of both sexes, we performed a physiological validation of a non-invasive method for evaluating adrenocortical activity in the Blue-fronted parrot by measuring glucocorticoid metabolites in droppings. It was possible to demonstrate that increased adrenocortical activity is well reflected in GCM measured in parrots with a cortisone EIA (detecting GCM with a 3,11-dione structure). Based on observed diurnal variations in GCM concentrations, time of the day has to be taken into consideration when planning experiments. This indispensable validation [34] provides the basis for a reliable non-invasive tool for further investigations to evaluate stress and its consequences in this bird species.

Unlike previous studies in birds (e.g., chickens [32]; capercaillie [40]; owls [41]), and mammals (Syrian hamster [42]; rat [43]; mouse [44]), the results of experiment 1 showed no sex differences in GCM concentrations in the Blue-fronted parrots. However, such a lack of sex differences was also found in other avian (e.g., Arctic passerine—*Zonotrichia leucophrys gambelii*—[45]), and mammalian (e.g, South American camelids [46]; guinea pig [47]) species underlining the need for careful validation.

The presence of inter-individual differences observed in our study regarding baseline (but also stress induced) GCM concentrations was found in almost all studies reported so far [21–23, 34]. Although parrots were housed at CEMPAS for at least six months before the beginning of the pre-experimental phase, their different previous life histories may have contributed to this variation. Furthermore different personalities (coping styles) may also contribute: For example, proactive individuals were reported to have lower HPA axis reactivity than reactive ones [48–50]. Whatever the reasons (for a discussion see also [28,34,51]), individual differences confirm the importance of the longitudinal study model (cross-over design) used in experiment 2 in which each animal is its own control [23], which is almost impossible to achieve in small birds if blood is sampled. Besides, individual variations in GC concentrations were also reported in blood samples (e.g. in cattle they were about 10 times greater than their GCM counterparts) which are only point in time estimates in contrast to faecal samples where concentrations are pooled over a certain period [28].

Moreover, there are other important findings in our study. In experiment 1 we observed a robust daily rhythm pattern of GCM excretion, suggesting the existence of a diurnal variation of plasma corticosterone in the Blue-fronted parrots. A GCM peak was detected in the samples collected between 5 to 11 a.m. This increase was detected before the onset of the daily photoperiod. There was a fast decline afterwards; so low concentrations were observed during the major fraction of the day. This pattern of GCM excretion in bird droppings was also observed in great tits (*Parus major*) [52] and geese [53], but not in galliformes, like chickens [32], quail [54], capercaillies (*Tetrao urogallus*) [55], and black grouse (*Tetrao tetrix*) [56]. The same daily rhythm pattern of GCM excretion found in experiment 1 was also observed in the females of the control treatments of experiment 2. Thus, this rhythmicity seems to be robust in the Blue-fronted parrots.

Reported gut passage times in Amazon parrots are about 2.1–5.5 h [57]. Considering this delay in faecal GCM excretion and the results of experiment 2, we assume that in parrots the plasma corticosterone peak occurs before the end of the dark phase at the time when animals wake up and start to increase their activity. These results are in agreement with previous observations in passerine birds in which there is a distinct unimodal daily rhythm with more corticosterone being released at the end of the dark phase [58–60].

On the other hand, additional physiological aspects should be taken into account in studies investigating diurnal variations of dropping GCM. Gastrointestinal motility also shows diurnal variations in avian species [61]. In mice, Touma et al. [44] found a faster GCM excretion during their active period. Different gastrointestinal motility may influence dropping rate in parrots. However, in our experiments with a large number of birds only in a few intervals (2.3%) no sample could be collected. Whatever contributed to diurnal changes in GCM, knowing their presence is important for setting up well designed experiments using non-invasive methods to evaluate adrenocortical activity.

Control and saline treatments of experiment 2 showed the same pattern of GCM excretion and the results were similar to the ones observed in experiment 1. This reinforces the importance of the 30-day adaptation period that birds were submitted in order to become adapted to the experimental conditions, as during this experimental phase all birds were in the same place and observed and heard each other during the experimental procedures.

Even considering the physical restraint and the pain associated with the injection procedure, saline treatment did not influence the GCM excretion and the concentrations were similar to the ones observed in the control group (*P* > 0.5). Since physical restraint is considered a stressful event in birds [62], our results strongly suggest that adult parrots previously accustomed to physical restraint by a specific person have lower plasma corticosterone when subjected again to the same procedure by the same person than those restrained for the first time and/or by an unfamiliar person. This is underlined by findings of Collete et al. [62] who observed lower corticosterone concentrations in *Amazona amazonica* parrots subjected to daily handling from 25 days of age until 38 days post-fledging compared to those that experienced the handling by the first time at age of 66 days. They concluded that daily neonatal handling permitted birds to be held at fledging without interpreting this as stressful.

The most efficacious stimulator of Psittacine corticosterone secretion is ACTH. Depending on the dose and route of administration, in general, ACTH causes a peak response in plasma corticosterone concentrations within 30 to 60 min after injection with a return to baseline concentrations within 120 to 240 min. For healthy cockatoos, macaws, Amazon parrots, and lorikeets, these mean peak corticosterone concentrations were four to 14 times higher than baseline concentrations [17,63,64].

As previously described in chicken [32], quail [54], capercailles [55], and black grouse [56], the group-specific cortisone enzyme immunoassay (EIA) was also suited to monitor GCM concentrations in droppings of the Blue-fronted parrots. Following ACTH injection, GCM concentrations increased and reached a peak after 3–9 h, and then dropped to pre-treatment concentrations. This finding is in agreement with the temporal pattern of excretion in other bird species such as male budgerigars [30], chickens [26,32], spotted owls [65], and black grouse [56]. The increase (about 13 times) after stimulation by ACTH was higher than reported for budgerigar parrots [30], and chicken [32], which suggests that this method should also be suited to detect minor stressors [34].

The stress-response coordinated by the hypothalamic-pituitary-adrenal (HPA) axis is highly conserved across all vertebrate taxa. Generally, glucocorticoids exert a negative feedback at all levels of the HPA axis, decreasing the release of ACTH and, consequently, circulating glucocorticoids [18,22,66]. However, in our study there was a transient increase in GCM concentrations 3 h after dexamethasone administration in males, and no effect in females. The response in males was similar to the one reported by Dehnhard et al. [26] who observed a transient increase in plasma corticosterone one hour after dexamethasone injections in chickens. However these same authors and Rettenbacher et al. [32], also working with chickens, could not detect increases in droppings GCM. On the other hand, a small increase in faecal GCM was also found in ruminants after dexamethasone injection [23].

Several studies demonstrated that doses of or above 1 mg dexamethasone per kg body weight are effective in inducing a decrease of plasma corticosterone concentrations in pigeons (*Columba livia domestica*) [67], chukar (*Alectoris chukar*) [37], and song sparrow (*Melospiza melodia*) [38]. However, like Dehnhard et al. [26] and Rettenbacher et al. [32] who used 2 mg/kg dexamethasone in chickens, we did not find a suppression of HPA axis activity in parrots, with 1 mg/kg dexamethasone based on GCM concentrations as these remained quite similar to those observed after control and saline treatment. Rettenbacher et al. [32] postulated that this may be due to the mineralocorticoid effects of corticosterone in chickens that are needed to maintain homeostasis of minerals. These results raise questions about possible species-specific responses of HPA axis to increased plasma glucocorticoids concentrations and their effects in the homeostasis in parrots and signalize the necessity of further studies. On the contrary, results may just indicate that the administered dose was too low to cause suppression.

Taken together, our results demonstrate that GCM, assayed with the group-specific cortisone EIA, accurately reflect a pharmacological stimulation of adrenocortical activity and dexamethasone (at a dose of 1 mg/kg) did not result in a distinct suppression of adrenocortical activity in the Blue-fronted parrots. As our parrots were kept at a stable, standardized condition, further research is needed to evaluate the influence of other possible confounding factors (such as free-ranging conditions, age, and reproductive stage of the birds, changing diet, season etc.) on GCM concentrations.

Based on our physiological validation, we demonstrated for the first time the suitability of an EIA to non-invasively monitor increases of adrenocortical activity in the Blue-fronted parrots. This method opens up new perspectives for stress and welfare investigations in this frequently kept bird species, which could exhibit extreme psychological and behavioural disorders in captivity. It could also help to detect poor welfare before disorders set in and for a better management of social groupings and breeding efforts.

## Supporting Information

S1 Dataset Diurnal variation of glucocorticoid metabolites in droppings of Blue-fronted parrots. (XLS)

S2 Dataset Glucocorticoid metabolites in droppings of female Blue-fronted parrots—Physiological validation. (XLSX)

S3 Dataset Glucocorticoid metabolites in droppings of male Blue-fronted parrots—Physiological validation. (XLSX)
